# Blood culture collection technique and pneumococcal surveillance in Malawi during the four year period 2003–2006: an observational study

**DOI:** 10.1186/1471-2334-8-137

**Published:** 2008-10-14

**Authors:** Neema Mtunthama, Stephen B Gordon, Temwa Kusimbwe, Eduard E Zijlstra, Malcolm E Molyneux, Neil French

**Affiliations:** 1 Malawi-Liverpool-Liverpool Wellcome Trust Laboratories, Blantyre, Malawi; 2 Liverpool School of Tropical Medicine, Liverpool, UK; 3 Department of Medicine, College of Medicine, Blantyre, Malawi; 4 London School of Hygiene & Tropical Medicine, London, UK; 5 Deceased

## Abstract

**Background:**

Blood culture surveillance will be used for assessing the public health effectiveness of pneumococcal conjugate vaccines in Africa. Between 2003 and 2006 we assessed blood culture outcome and performance in adult patients in the central public hospital in Blantyre, Malawi, before and after the introduction of a dedicated nurse led blood culture team.

**Methods:**

A prospective observational study.

**Results:**

Following the introduction of a specialised blood culture team in 2005, the proportion of contaminated cultures decreased (19.6% in 2003 to 5.0% in 2006), blood volume cultured increased and pneumococcal recovery increased significantly from 2.8% of all blood cultures to 6.1%. With each extra 1 ml of blood cultured the odds of recovering a pneumococcus increased by 18%.

**Conclusion:**

Standardisation and assessment of blood culture performance (blood volume and contamination rate) should be incorporated into pneumococcal disease surveillance activities where routine blood culture practice is constrained by limited resources.

## Background

Blood cultures are an essential component of good clinical care in the diagnosis and management of blood stream infections (BSI) which are frequent in hospitalised patients in Malawi and the rest of Africa [1-6]. Information from blood culture surveillance is also an important tool for establishing public health priorities, assessing the impact of interventions – particularly vaccines – and for providing information on antimicrobial resistance patterns to help formulate prescribing guidelines for empirical therapy. Blood culture surveillance has been the key tool used in the USA for recognising the enormous potential of childhood pneumococcal conjugate vaccination for decreasing disease in adults[7].

In Malawi as in much of the rest of sub-Saharan Africa, blood culture facilities are available in only a limited number of centres. As a consequence single reports are often extrapolated as representative of disproportionately large regions and time periods, and inaccuracies in these reports are more likely to be perpetuated as a result of the lack of alternative data. Blood cultures need to be specific and sensitive and therefore as representative as possible of the true BSI disease burden and particularly so where BSI surveillance is restricted to very few sites.

Isolation of bacteria from blood is usually taken as definitive proof of disease aetiology; this is particularly true for pneumococcal pneumonia where blood culture remains the gold-standard for diagnosis. However blood cultures lack sensitivity, which may vary as a result of both laboratory and clinical factors. Laboratory factors include choice of culture media, speed of incubation and duration of incubation. Among clinical factors recent receipt of antibiotics and poor collection technique leading to contamination decreases sensitivity, while culturing larger volumes of blood generally increases the yield of positive results [8-13]. Investigation of the relationship of blood volume to recovery has tended to concentrate on large volumes of blood in excess of 20 mls and not on the relatively smaller volumes most typically taken during routine clinical practice in Africa.

The blood culturing service at the Queen Elizabeth Central Hospital (QECH) in Blantyre, Malawi is the only large scale, unselected and continuous assessment of BSI in Malawi and thus provides information of national importance to health planners and researchers. Samples for blood culture have traditionally been taken by nurses, medical and paramedical staff in attendance on the wards of the QECH. In March 2003 an audit of blood culture performance was carried out in the medical wards, revealing poor collection technique and inadequate volumes of collected blood. This information was fed back to staff starting in January 2004 with regular updates throughout that year but with little overall change in the outcome (contamination rate and isolation rate) of blood cultures. In January 2005 a dedicated team of nurses was established to perform all the blood cultures in adult patients in the QECH. This was a direct consequence of the failure to measure an improvement in blood culture performance through feedback to the general clinical staff. We report the changes in blood culture results following the introduction of this team and relate bacterial isolation to blood volume collected.

## Methods

All of the work described has been carried out in the adult medical wards of the Queen Elizabeth Central hospital (QECH) between January 2003 and December 2006. The QECH is the largest public hospital in Malawi and provides care for the more than one million residents in Blantyre district and takes referrals from the southern region of the country. The medical department has ~250 beds occupied at any given time and admits ~10,000 patients annually.

In February-April 2003 an audit of blood culture technique and volume was undertaken. In January 2004 the results of this audit were fed back to staff involved in blood culture collection. Throughout 2004 there were regular updates of blood culture performance provided at least monthly at medical department meetings and including staff responsible for taking blood for culture. When new staff arrived in the department, demonstrations were given at three monthly intervals by study team members on proper blood collection technique. In January 2005 a dedicated team of nurses was introduced with responsibility to perform all blood cultures. Nurses were chosen as no cadre of staff exist in Malawi with a license to perform phlebotomy. These nurses were given appropriate training including laboratory visits and are supervised by a senior nurse manager. Whilst they also undertake general nursing duties their work priority is the performance of blood cultures. They take responsibility for the management of physical resources required for phlebotomy and liaison with the laboratory. If they encounter a problem they have direct access to the nurse manager. The results of blood cultures are returned to patients and their medical team through the blood culture nurses. Targets for contamination rates are set at 5%.

Blood cultures are requested by the admitting medical officer. The majority of these staff change at regular intervals after a few months in post but are encouraged to follow departmental guidelines for ordering of blood cultures. These include axillary temperature > 37.5°C or < 35.0°C, clinical syndrome of pneumonia, sepsis or meningitis, any altered level of consciousness or any life threatening illness as judged by the admitting officer. These policies and guidelines for the selection of patients for blood culture did not change throughout the period of the present study. All departmental staff receive a small printed copy of departmental protocols to be carried on their person for reference and undergo a period of training at the commencement of their attachment. There is no regular formal audit of compliance with protocol.

Blood culture analysis is performed using an automated system (BacTalert^®^, Bio-Merieux). Blood is removed from the patient and transferred to a single aerobic blood culture bottle containing the manufacturer's standard culture medium. Bottles are either placed in a ward-based incubator or transferred immediately to the microbiology laboratory for processing, depending on the time of collection. A count of blood culture bottles provided by the lab is kept and compared to blood culture bottles received back from the wards along with reports of bottle spoilage. This process ensures all blood cultures taken are analysed.

Processing of samples follows standard operating procedures consistent with accepted laboratory practice. These protocols have been unaltered over the four years of the surveillance period. Isolation of Diphtheroids, coagulase negative Staphylococci, Micrococci spp or Bacillus spp other than *anthracis *is recorded as contaminants. All other isolates are treated as significant.

Collected blood volume was measured during Feb/March, 2003 and Feb/March 2005. All blood cultures arriving in the laboratory on Mondays and Thursdays were subjected to assessment. Weight was measured by subtracting the mean unfilled and uncapped blood culture bottle weight from the weight of bottles delivered to the laboratory. The mean weight of an unused bottle was estimated by sequential measurements on 20 unused bottles. Weights of unused bottles were highly consistent within each period of assessment: in 2003 mean weight was 93.7 g, (SD 0.15) and in 2005 it was 68.4 g, (SD 0.16) (blood culture bottle structure was changed during the intervening period by the manufacturer). The staff in both the hospital and laboratory were unaware that an assessment of blood culture volumes was underway. In 2003 we assessed 500 bottles; in 2005 we measured 250 bottles. This would provide sufficient power to demonstrate a significant increase in blood volume above a geometric mean of 5 mls.

Results of blood cultures are recorded in a Microsoft Access^® ^database. Statistical analysis was performed using STATA8 software (College Station, Texas). Comparison of blood volumes was performed using the unpaired Student's t-test, the relationship between blood volume collected and isolation rate was performed using logistic regression analysis. Temporal changes in isolate recovery were evaluated by comparing recovery rates in the pre-blood culture nurse service years 2003/4 to the post blood culture service years 2005/6 using the chi^2 ^test.

No ethical approval was sought for this study as it used routinely collected data. Analysis was undertaken on an anonymised database.

## Results

There was no significant change between 2003 and 2004 following the introduction of performance feedback to staff (Table 1). Following the introduction of the blood culture team in 2005, there was a significant decline in the rate of retrieval of contaminants from blood cultures, from 19.6% to 5.0% (Table 1). The quantity of blood collected for culture also significantly increased during this period, from a median of 4.6 to 9.7 mls per bottle (Table 1).

**Table 1:** Total number of blood cultures taken and analysed over the four years 2003–2006 inclusive and the proportion of cultures growing a contaminant or significant isolate

**Year**	**2003**	**2004**	**2005**	**2006**	**P**^1^	**OR**^2^
**Total blood cultures**	7141	5709	6129	5650		
**Contaminants **n(%)	1402(19.6)	1071(18.8)	442(7.2)	280(5.0)	< 0.01	0.27
**All significant isolates**	1267(17.7)	931(16.3)	1203(19.6)	1020(18.1)	< 0.01	1.13
***S. pneumoniae***	207(2.9)	152(2.7)	410(6.7)	310(5.5)	< 0.01	2.26
**non-typhoidal Salmonella**	758(10.6)	483(8.5)	480(7.8)	374(6.6)	< 0.01	0.73
***C. neoformans***	64(0.9)	53(0.9)	73(1.2)	118(2.1)	< 0.01	1.79
***E. coli***	93(1.3)	76(1.3)	114(1.9)	115(2.0)	< 0.01	1.48
**Klebsiella spp**	25(0.4)	24(0.4)	13(0.2)	12(0.2)	0.02	0.55
***S. aureus***	38(0.5)	43(0.8)	26(0.4)	16(0.3)	< 0.01	0.56
**Group A streptococcus**	10(0.1)	12(0.2)	15(0.2)	15(0.3)	0.15	
***S. typhi***	3(0.0)	3(0.1)	8(0.1)	11(0.2)	< 0.01	3.46
**Other Gram positive**^3^	19(0.3)	34(0.6)	27(0.4)	16(0.3)	0.55	
**Other Gram negative**^4^	50(0.7)	51(0.9)	37(0.6)	33(0.6)	0.07	

**Median volume ml (IQR)**	4.6(0.3–10.1)		9.7(1.9–13.3)^2^		< 0.001	

^1 ^The p value has been derived by a two-way comparison comparing 2003/4 (pre-dedicated culture service) vs 2005/6 (post dedicated service) using a chi^2 ^test. ^2 ^The odds ratio of recovery during the 2005/6 blood culture intervention year compared to the pre-blood culture service period. ^3 ^Other Gram positive isolates were primarily non-pneumococcal alpha haemolytic Streptococci (72 of 96, 75%) ^4 ^Other Gram negative isolates included Haemophilus spp. (16 of 171, 9.4%), Pseudomonas spp. (18, 10.5%), Neisseria meningitidis (14, 8.2%), Acinetobacter spp. (32, 18.7%), Enterobacter spp. (38, 22.4%) and other coliforms (53, 31.0%).

Non-typhoidal Salmonella and *Streptococcus pneumonia *were the most frequently isolated organisms during all three periods and made up 76.2, 68.2, 74.0 and 67.1% of all significant isolates during the four study years respectively. The other major isolates are detailed in Table 1. The increased recovery of *S. pneumoniae *between 2003/4 and 2005/6 was highly significant with a 118% increase in isolation. There was also a significant 25% decrease in the recovery of non typhoidal Salmonella during these two periods (p < 0.001), (Table 1). Amongst other isolates there was a significant increase in recovery of *E. coli, Cryptococcus neoformans *and *Salmonella typhi*.

Geometric mean blood volumes were highest in blood cultures from which *S. pneumoniae *and *C. neoformans *were recovered (Table 2). There was also a significant increasing chance of recovering *S. pneumoniae *with larger blood volumes cultured. Over the range of blood volumes collected in this study, for every extra 1 ml of blood the odds of recovering *S. pneumoniae *increased by 18%. This trend was not seen for non typhoidal Salmonella. When adjusted for year of recovery this finding persisted. There was a trend for decreasing recovery of both other Gram-negative and Gram-positive isolates with increased blood volume. However the majority of these isolates were recorded during the 2003 assessment period (35 vs 2) which limits the validity of this comparison.

**Table 2:** Geometric mean blood volume cultured in relation to isolate recovery for 760 blood cultures assessed during the two surveillance periods in 2003 and 2005

	N	Mean volume ml	95% CI	OR (95%CI)	OR^a ^(95%CI)
**No growth**	566	5.51	5.28–5.75		
**Contaminants**	52	3.76	3.17–4.45		
***S. pneumoniae***	31	6.71	5.69–7.93	1.18 (1.05–1.33)	1.29 (1.08–1.53)
**Non typhi *Salmonella***	70	5.21	4.61–5.87	0.99 (0.90–1.07)	0.97 (0.86–1.09)
***Cryptococcus neoformans***	4	6.72	2.45–18.45	1.19 (0.86–1.65)	0.86 (0.61–1.20)
**Other Gram negative**	27	3.04	1.99–4.65		
**Other Gram positive**	10	3.70	2.63–5.20		

For clarity, Gram negative and Gram positive bacteria other than *S. pneumoniae *and the non-typhi Salmonella have been grouped together. Odds Ratios are presented unadjusted (OR) and adjusted (OR^a^) for year of sampling and represent the chances of recovering an isolate with each extra ml of blood over the range of volumes taken in this study 1–13 mls.

## Discussion

The QECH is an important tertiary referral centre in Malawi providing a blood culture service for patient care. The results of blood cultures from this institution have been reported in the past[6,14] and have helped determine clinical and research priorities and antibiotic prescribing practice. Inadequacies of technique and procedure in the sampling of blood for culture were corrected during 2005 by the introduction of a team of blood culture nurses. As a result of this we measured an increased yield of blood culture isolates, an effect that was most dramatic with the isolation of pneumococci. Our findings suggest that surveillance for invasive pneumococcal disease in our region should pay close attention to the quality and quantity of blood collection at the bed-side.

The potential for selection bias in this study was low. Blood cultures were ordered as a part of standard departmental clinical care and unconnected with any of the investigative team. Departmental indications for blood cultures remained unchanged over the course of the study. The proportion of all adult admissions sampled each year remained relatively constant around 60% (data not shown) suggesting that there was no fundamental change in the individuals sampled that might bias sampling towards respiratory illness and greater recovery of pneumococcus. Over the four year period, data from active surveillance on clinical presentation to hospital indicate no secular trends in illness presentation in this population. Inoculated blood volumes were assessed on a representative sample of blood cultures. Although the sample selection was not random the sampling method was unlikely to lead to any systematic bias in isolate recovery or volume between the two assessment periods: clinical staff were unaware of the blood culture volume assessment; samples were taken from acute admissions; and as noted above, there was no evidence of a major change in pattern of clinical presentation during this period. Laboratory practice was consistent throughout the duration of the study.

The principal weakness of the study is the historical nature of the comparison. We are unable to fully exclude the possibility of temporal shifts in the pattern of pneumococcal disease and other significant isolates, or of changes in the pattern of health seeking behaviour and population. Pneumococcal disease does show seasonal variation[3,15]. By assessing blood volumes over similar seasons in the two assessment years, we have minimised the possible confounding effect of seasonal changes on the relationship between sample volume and retrieval of *S pneumoniae*. Longer term changes i.e. the possibility of a steadily increasing burden of pneumococcal infection in the community, are less easy to control for. Accessibility to antiretrovirals (ART) for the HIV-infected and improvements in health care access may have altered health seeking patterns. There is no information from this region to know if this is the case, although it is unlikely that this would easily explain the rise in pneumococcal isolation indeed access to ART might be expected to reduce disease burden[16] and the number of health service facilities in the region has remained the same over the period of the study. Population size and structure within the hospital catchment area is also subject to demographic change. However during the period of the study there has been no major population shifts with annual growth estimated at 2.3%[17]. Urbanisation in Malawi is relatively slow compared to other African countries and the prevalence of adult HIV in this region has remained stable at around 12% which would suggest there has been no large influx of susceptible individuals as an explanation for the increased numbers of cases. There is no evidence to support a fundamental change in the behaviour and biology of *S. pneumoniae*. Serotype distribution has remained relatively constant over several years [18] with an absence of any large epidemics of serotypes 1,3,5 or 12 which have been associated with large outbreaks in sub-Saharan Africa and elsewhere. Furthermore the demonstrated association between recovery, blood volume and reduced contamination provides support to the view that increased recovery is a function of sampling and not an epidemic phenomenon.

The decrease in recovery of non typhoidal Salmonella is less easy to explain. Salmonellae are potential skin contaminants in our setting as well as invasive pathogens in their own right. Reduced skin contamination through improved skin cleansing is probably the explanation for the reduced recovery of *Staphylococcus aureus *and may also be the case for our reduced recovery of other Gram negatives. However temporal shifts in disease burden need to be considered [19]. In the two years preceding this report (2001 and 2002) recovery of non-typhoidal Salmonellae and *S. pneumoniae *were recorded at a similar rate to that recorded in 2003 (Salmonellae 8.0% and 10.1%, *S. pneumoniae *2.2% and 2.5% respectively, unpublished data, blood culture analysis using a manual method), which suggests that temporal changes take place gradually over long periods of time and that the findings reported in 2003 and 2004 are not unique in terms of recovery rates of significant isolates. Increasing use of anti-retroviral drugs during 2005 and 2006 may have reduced the incidence of NTS bacteraemia in the adult population in Blantyre. Longer term surveillance incorporating other centres will be important to understand the evolving epidemiology of BSI preferably nested in settings subjected to demographic surveillance.

The association between increased blood volume and improved recovery of pneumococcus is not in itself surprising. Past studies and accepted knowledge support the view that greater volumes of blood improve the recovery of microorganisms[8-13,20], although there are exceptions to this[21]. Similarly it is not surprising that there should be a different relationship between volume and isolation rate for different bacteria. The quantity of bacteria in a given quantity of blood, the viability of these bacteria and their robustness during venesection, inoculation, transportation, incubation and analysis will all play a part. The failure to see increased recovery of non-typhoidal salmonella with larger blood culture volumes may reflect the ease of recovery of this organism from individuals with advanced HIV, who are unable to control the bacteraemia[22].

What is surprising from this study is the extent of the change in recovery of pneumococci in relation to the other principal blood culture isolates over the range of blood volumes suitable for inoculation into a single blood culture bottle. Single bottle inoculation has been adopted as a routine as it minimises costs and also fits with local cultural concerns over removal of blood. This will be typical of many resource-limited settings. Our findings are very different from a previous report in the USA that recorded no change in the recovery of Gram positive organisms using the BacTalert^® ^system comparing 5 and 10 ml inoculums[23]. This suggests a fundamentally different host response to bacteraemia in our patient population which alters bacterial recovery and the high prevalence of HIV co-infection in over 70% of adult in-patients is likely to be the major determining factor. Pneumococcal bacteraemia is known to be more common during respiratory infection in HIV-infected adults compared to the HIV-uninfected[24,25]. A mechanism to explain a differing volume/recovery relationship in the presence of HIV is however not currently known.

## Conclusion

These findings suggest we should be cautious about the way we interpret and use data from routine blood culture services where there are severe resource limitations. Uncritical reporting may falsely skew and under-report blood stream infections, especially *S. pneumoniae *bacteraemia. There are rapidly evolving initiatives to introduce pneumococcal conjugate vaccines into the vaccination schemes in developing nations. An important component of this introduction will be the availability of reliable pneumococcal disease surveillance for the monitoring of both direct and indirect benefits, and serotype changes induced by the vaccine. These findings suggest that to achieve accurate results staff should be educated but most importantly monitored and encouraged through an appropriate line of management. The failure to see improvements in blood culture performance during 2004 we believe reflects the low priority given by staff to the performance of blood cultures, the difficulties in finding resources and lack of encouragement and feedback to first-contact clinical staff from their immediate line managers. By having a small team of nurses, closely monitored, in daily communication with the laboratory and nurse manager and working towards targets, performance was improved. The resources to undertake work were also more easily controlled and always available. We conclude by suggesting that a minimum set of standards required for adequate surveillance should be established. These should incorporate an audit of blood culture quality and blood volume collection, and a clear line of responsibility for supervising blood culture collection suitable for local circumstances in order to enhance the accuracy and reliability of collected data.

## Competing interests

The authors declare that they have no competing interests.

## Authors' contributions

NM and NF conceived the study. NM, TK, MK undertook the study and data collection. NM produced the first draft of the article. EZ, MM, SB provided scientific comment and support. All authors contributed to subsequent drafts and with the exception of TK have reviewed and approved the submitted version. TK died prior to the final draft of the paper.

## Pre-publication history

The pre-publication history for this paper can be accessed here:



## References

[B1] Ssali FN, Kamya MR, Wabwire-Mangen F, Kasasa S, Joloba M, Williams D, Mugerwa RD, Ellner JJ, Johnson JL (1998). A prospective study of community-acquired bloodstream infections among febrile adults admitted to Mulago Hospital in Kampala, Uganda. J Acquir Immune Defic Syndr Hum Retrovirol.

[B2] Archibald LK, McDonald LC, Nwanyanwu O, Kazembe P, Dobbie H, Tokars J, Reller LB, Jarvis WR (2000). A hospital-based prevalence survey of bloodstream infections in febrile patients in Malawi: implications for diagnosis and therapy. J Infect Dis.

[B3] Bell M, Archibald LK, Nwanyanwu O, Dobbie H, Tokars J, Kazembe PN, Reller LB, Jarvis WR (2001). Seasonal variation in the etiology of bloodstream infections in a febrile inpatient population in a developing country. Int J Infect Dis.

[B4] Gordon MA, Walsh AL, Chaponda M, Soko D, Mbvwinji M, Molyneux ME, Gordon SB (2001). Bacteraemia and mortality among adult medical admissions in Malawi – predominance of non-typhi salmonellae and Streptococcus pneumoniae. J Infect.

[B5] Arthur G, Nduba VN, Kariuki SM, Kimari J, Bhatt SM, Gilks CF (2001). Trends in bloodstream infections among human immunodeficiency virus-infected adults admitted to a hospital in Nairobi, Kenya, during the last decade. Clin Infect Dis.

[B6] Peters RP, Zijlstra EE, Schijffelen MJ, Walsh AL, Joaki G, Kumwenda JJ, Kublin JG, Molyneux ME, Lewis DK (2004). A prospective study of bloodstream infections as cause of fever in Malawi: clinical predictors and implications for management. Trop Med Int Health.

[B7] Whitney CG, Farley MM, Hadler J, Harrison LH, Bennett NM, Lynfield R, Reingold A, Cieslak PR, Pilishvili T, Jackson D, Facklam RR, Jorgensen JH, Schuchat A (2003). Decline in invasive pneumococcal disease after the introduction of protein-polysaccharide conjugate vaccine. N Engl J Med.

[B8] Hall MM, Ilstrup DM, Washington JA (1976). Effect of volume of blood cultured on detection of bacteremia. J Clin Microbiol.

[B9] Sandven P, Hoiby EA (1981). The importance of blood volume cultured on detection of bacteraemia. Acta Pathol Microbiol Scand [B].

[B10] Plorde JJ, Tenover FC, Carlson LG (1985). Specimen volume versus yield in the BACTEC blood culture system. J Clin Microbiol.

[B11] Mermel LA, Maki DG (1993). Detection of bacteremia in adults: consequences of culturing an inadequate volume of blood. Ann Intern Med.

[B12] Li J, Plorde JJ, Carlson LG (1994). Effects of volume and periodicity on blood cultures. J Clin Microbiol.

[B13] Brown DR, Kutler D, Rai B, Chan T, Cohen M (1995). Bacterial concentration and blood volume required for a positive blood culture. J Perinatol.

[B14] Gordon MA, Walsh AL, Chaponda M, Soko D, Mbvwinji M, Molyneux EM, Gordon SB (2001). Bacteraemia and mortality among adult medical admissions in Malawi – predominance of non-*typhi Salmonellae *and *Streptococcus pneumoniae*. J Infect.

[B15] Gordon SB, Walsh AL, Chaponda M, Gordon MA, Soko D, Mbwvinji M, Molyneux ME, Read RC (2000). Bacterial meningitis in Malawian adults: pneumococcal disease is common, severe, and seasonal. Clin Infect Dis.

[B16] Heffernan RT, Barrett NL, Gallagher KM, Hadler JL, Harrison LH, Reingold AL, Khoshnood K, Holford TR, Schuchat A (2005). Declining incidence of invasive Streptococcus pneumoniae infections among persons with AIDS in an era of highly active antiretroviral therapy, 1995–2000. J Infect Dis.

[B17] UNICEF Unite for children. http://www.unicef.org/infobycountry/malawi_statistics.html.

[B18] Gordon SB, Kanyanda S, Walsh AL, Goddard K, Chaponda M, Atkinson V, Mulwafu W, Molyneux EM, Zijlstra EE, Molyneux ME, Graham SM (2003). Poor potential coverage for 7-valent pneumococcal conjugate vaccine, Malawi. Emerg Infect Dis.

[B19] Gordon MA, Graham SM, Walsh AL, Wilson L, Phiri A, Molyneux E, Zijlstra EE, Heyderman RS, Hart CA, Molyneux ME (2008). Epidemics of invasive Salmonella enterica serovar enteritidis and S. enterica Serovar typhimurium infection associated with multidrug resistance among adults and children in Malawi. Clin Infect Dis.

[B20] Arpi M, Bentzon MW, Jensen J, Frederiksen W (1989). Importance of blood volume cultured in the detection of bacteremia. Eur J Clin Microbiol Infect Dis.

[B21] Archibald LK, Dobbie H, Kazembe P, Nwanyanwu O, McKnight C, Byrne T, Addison RM, Bell M, Reller LB, Jarvis WR (2001). Utility of paired BACTEC MYCO/F LYTIC blood culture vials for detection of bacteremia, mycobacteremia, and fungemia. J Clin Microbiol.

[B22] Gordon MA, Banda HT, Gondwe M, Gordon SB, Boeree MJ, Walsh AL, Corkill JE, Hart CA, Gilks CF, Molyneux ME (2002). Non-typhoidal salmonella bacteraemia among HIV-infected Malawian adults: high mortality and frequent recrudescence. AIDS.

[B23] Weinstein MP, Mirrett S, Wilson ML, Reimer LG, Reller LB (1994). Controlled evaluation of 5 versus 10 milliliters of blood cultured in aerobic BacT/Alert blood culture bottles. J Clin Microbiol.

[B24] Koulla-Shiro S, Kuaban C, Belec L (1996). Acute community-acquired bacterial pneumonia in Human Immunodeficiency Virus (HIV) infected and non-HIV-infected adult patients in Cameroon: aetiology and outcome. Tuber Lung Dis.

[B25] Janoff EN, Rubins JB (1997). Invasive pneumococcal disease in the immunocompromised host. Microb Drug Resist.

